# Poly[hydrazin-1-ium [diaqua­bis­(μ_4_-pyridazine-3,6-dicarboxyl­ato)trilithate] monohydrate]

**DOI:** 10.1107/S1600536812012743

**Published:** 2012-03-31

**Authors:** Wojciech Starosta, Janusz Leciejewicz

**Affiliations:** a Institute of Nuclear Chemistry and Technology, ul. Dorodna 16, 03-195 Warszawa, Poland

## Abstract

The structure of the title compound, {(N_2_H_5_)[Li_3_(C_6_H_2_N_2_O_4_)_2_(H_2_O)_2_]·H_2_O}_*n*_, is composed of mol­ecular dimers, each built up of two symmetry-related Li^I^ ions with distorted trigonal–bipyramidal coordinations bridged by two deprotonated ligand mol­ecules *via* their *N*,*O*-bonding sites. Doubly solvated Li^I^ ions with a distorted tetra­hedral geometry link adjacent dimers, forming a polymer generated by bridging bidentate carboxyl­ato O atoms to Li^I^ ions in adjacent dimers, forming anionic layers parallel to the *ac* plane with monoprotonated hydrazinium cations and crystal water mol­ecules positioned between them. The layers are held together by an extended system of hydrogen bonds in which the hydrazinium cations and coordinated and crystal water mol­ecules act as donors and carboxyl­ate O atoms act as acceptors.

## Related literature

For the crystal structures of Li^I^ complexes with pyridazine-3,6-dicarboxyl­ate ligands, see: Starosta & Leciejewicz (2010 ▶, 2011 ▶, 2012 ▶). The structure of a hydrazine adduct of pyridazine-3,6-dicarb­oxy­lic acid was also reported by Starosta & Leciejewicz (2008 ▶).
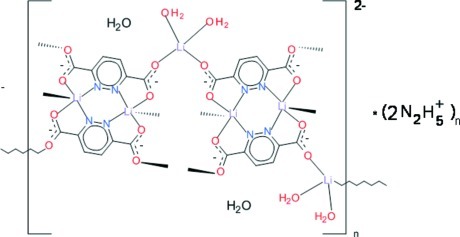


## Experimental

### 

#### Crystal data

(N_2_H_5_)[Li_3_(C_6_H_2_N_2_O_4_)_2_(H_2_O)_2_]·H_2_O
*M* *_r_* = 440.12
Triclinic, 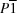

*a* = 5.215 (1) Å
*b* = 7.3356 (15) Å
*c* = 24.001 (5) Å
α = 97.62 (3)°
β = 90.62 (3)°
γ = 95.77 (3)°
*V* = 905.2 (3) Å^3^
*Z* = 2
Mo *K*α radiation
μ = 0.14 mm^−1^
*T* = 293 K
0.40 × 0.14 × 0.06 mm

#### Data collection

Kuma KM-4 four-cricle diffractometer
Absorption correction: analytical (*CrysAlis RED*; Oxford Diffraction, 2008 ▶) *T*_min_ = 0.982, *T*_max_ = 0.992
5141 measured reflections
4676 independent reflections
2660 reflections with *I* > 2σ(*I*)
*R*_int_ = 0.051
3 standard reflections every 200 reflections intensity decay: 1.4%

#### Refinement

*R*[*F* ^2^ > 2σ(*F* ^2^)] = 0.057
*wR*(*F* ^2^) = 0.243
*S* = 1.06
4676 reflections
329 parameters
10 restraints
H atoms treated by a mixture of independent and constrained refinement
Δρ_max_ = 0.56 e Å^−3^
Δρ_min_ = −0.48 e Å^−3^

### 

Data collection: *KM-4 Software* (Kuma, 1996 ▶); cell refinement: *KM-4 Software*; data reduction: *DATAPROC* (Kuma, 2001 ▶); program(s) used to solve structure: *SHELXS97* (Sheldrick, 2008 ▶); program(s) used to refine structure: *SHELXL97* (Sheldrick, 2008 ▶); molecular graphics: *SHELXTL* (Sheldrick, 2008 ▶); software used to prepare material for publication: *SHELXTL*.

## Supplementary Material

Crystal structure: contains datablock(s) I, global. DOI: 10.1107/S1600536812012743/kp2395sup1.cif

Structure factors: contains datablock(s) I. DOI: 10.1107/S1600536812012743/kp2395Isup2.hkl

Additional supplementary materials:  crystallographic information; 3D view; checkCIF report

## Figures and Tables

**Table 1:** Selected bond lengths (Å)

Li1—N11	2.260 (6)
Li1—O13	1.982 (6)
Li1—O11^i^	2.010 (5)
Li1—O12^ii^	2.088 (6)
Li1—N12^i^	2.144 (6)
Li2—O21	1.990 (6)
Li2—N21	2.186 (6)
Li2—O24^iii^	1.995 (6)
Li2—O22^iv^	2.097 (6)
Li2—N22^iii^	2.276 (6)
Li3—O14	1.893 (5)
Li3—O2	1.938 (6)
Li3—O1	1.952 (6)
Li3—O23	1.934 (6)

Symmetry codes: (i) 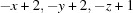; (ii) 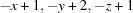; (iii) 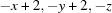; (iv) 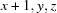.

**Table 2:** Hydrogen-bond geometry (Å, °)

*D*—H⋯*A*	*D*—H	H⋯*A*	*D*⋯*A*	*D*—H⋯*A*
N2—H3⋯O24^v^	0.89 (2)	1.95 (2)	2.824 (4)	168 (6)
N2—H5⋯O3^vi^	0.92 (2)	1.82 (3)	2.709 (5)	162 (7)
N1—H2⋯O23^vii^	0.90 (2)	2.07 (2)	2.965 (4)	175 (5)
N2—H4⋯O13^v^	0.90 (2)	1.96 (5)	2.716 (4)	140 (6)
N1—H1⋯O11^viii^	0.88 (2)	2.12 (2)	2.981 (4)	167 (5)
O2—H21⋯O1^iv^	0.82 (2)	1.94 (3)	2.737 (4)	163 (7)
O2—H22⋯O22^ix^	0.83 (2)	2.07 (3)	2.868 (4)	162 (7)
O3—H31⋯O12^viii^	0.84 (2)	1.93 (2)	2.741 (4)	164 (5)
O3—H32⋯O14	0.81 (2)	2.11 (3)	2.875 (4)	156 (5)
O1—H12⋯O21^ix^	0.93 (5)	1.76 (5)	2.684 (3)	174 (4)
O1—H11⋯N1	0.82 (5)	2.00 (5)	2.814 (4)	171 (5)

Symmetry codes: (iv) 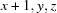; (v) 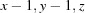; (vi) 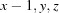; (vii) 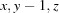; (viii) 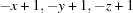; (ix) 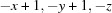.

**Fig. 1.**
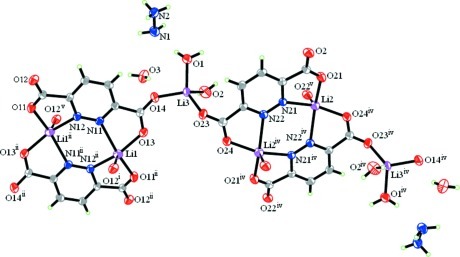
A molecule of the title compound with atom labelling scheme and 50% probability displacement ellipsoids. For clarity, hydrogen atoms are not labelled. Symmetry code: i-x + 1, -y + 2, -z + 1; ii -x + 2, -y + 2, -z + 1; iv -x + 2, -y + 2,-z; vx + 1, y, z.

**Fig. 2.**
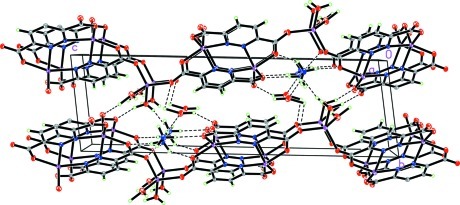
Packing diagram of the structure viewed along the a axis.

## supplementary crystallographic information

### Comment

It has been reported (Starosta & Leciejewicz, 2010), that the structure of a Li^I^ complex with pyridazine-3,6-dicarbaxylate and water ligands is built of centrosymmtric monomers bridged by strong, centrosymmetric hydrogen bonds. Deprotonization by an addition of a small amount of hydrazine resulted in a compound with a polymeric structure composed of centrosymmetric tetramers in which two Li^I^ ions, bridged by two fully deprotonated pyridazine-3,6-dicarboxylate ligands are linked to two triply solvated Li^I^ ions. The latter bridge the tetramer *via* two aqua O atoms to adjacent tetramers (Starosta & Leciejewicz, 2011). An addition of few more drops of hydrazine gave rise to a compound with a structure built of centrosymmetric anions in which two Li^I^ ions, are bridged by two fully deprotonated pyridazine-3,6-dicarboxylato ligands with the charge compensated by two mono protonated hydrazine cations and neutral centrosymmetric tetrameric molecules as in previous compound. While studying the reaction products of LiNO_3_ with the title ligand we have obtained a new compound. Its structure is reported below. The structure of the title compound is polymeric. Its unit cell contains two symmetry independent dinuclear moieties (dimers) related by a centre of symmetry each built of two Li ions and two fully deprotonated pyridazine-3,6-dicarboxylate ligands (Fig. 1). The dimers are bridged by a doubly solvated symmetry independent Li3 ion. In each dimer, ligand carboxylato groups act as bidentate. Apart from participation in the *N*,*O*-bonding groups chelating the intra-dimer Li ions, they use the second O atoms to bridge the dimers to doubly solvated Li3 ions forming an anionic ribbon propagating in the unit cell *c* direction (Symmetry code:^i^-*x* + 1, -*y* + 2, -*z* + 1; ^ii^ -*x* + 2, -*y* + 2, -*z* + 1; ^iv^ -*x* + 2, -*y* + 2,-*z*; ^v^*x* + 1, *y*, *z*)(Fig. 1). A second bridging pathway with a direction normal to the ribbons links them into a polymeric two-dimensional framework (Fig. 2). The two dimers and two doubly solvated Li3 ions carry a charge of (2-) which is compensated by two inversion symmetry related mono-protonated hydrazinium cations positioned between adjacent dimers. In the asymmetric unit there is a crystal water molecule. The coordination polyhedron around the Li1 ion, a distorted trigonal-bipyramid, is composed of O13, O12^i^, N12^ii^ atoms which make an equatorial plane, the Li1 ion is 0.0863 (2) Å out of it, N11 and O11^ii^ atoms are at apical positions. The strongly distorted trigonal-bipyramidal coordination of the Li2 ion consists of N21, O22^v^ and O24^iv^ atoms which constitute its equatorial plane with the Li2 ion 0.0893 (2) Å out of it; O21 and N22^iv^ atoms are at the apices. The penta-coordination mode of Li1 and Li2 ions can be also visualized as a transition from distorted trigonal-bipyramid to a deformed square-pyramid, the latter with O12^i^ and O24^iv^ atoms at apical positions in case of Li1 and Li2 ions, respectively. Li—O and Li—N bond distances (Table 1) fall in the same range as those observed in the Li^I^ complexes with title and water ligands (Starosta & Leciejewicz, 2010, 2011, 2012). The ligand 1 and 2 pyridazine rings are planar with r.m.s. of 0.0123 (2) Å and 0.0077 (1) Å, respectively. The carboxylato groups C17/O11/12 and C18/O13/O14 make dihedral angles with the ligand ring 1 of 17.8 (2)° and 13.3 (2)°, respectively; the respective angles between C27//O21/O22 and C28/O23/24 groups and the ligand ring 2 are 15.9 (2)° and 8.4 (2)°. Li3 ion shows a distorted tetrahedral coordination geometry with a pair of bridging carboxylato O13 and O23 atoms and a pair of coordinated water O1, O2 atoms in *trans* arrangement. Li3 ion is neither coplanar with the dimer 1 nor the dimer 2 as indicated by the O14—Li3—O23 angle of 110.6 (3)°; its position in respect to adjacent dimers makes a half-open cavity which is occupied by the hydrazinium cation. The layers are held together by an extended system of hydrogen bonds in which hydrazinium cation, coordinated, and crystal water molecules act as donors to carboxylato O atoms (Table 2).

### Experimental

A reaction of 1 mmol of pyridazine-3,6-dicarboxylic acid with 2 mmol s of lithium nitrate both dissolved in 50 ml of hot water and then boiled under reflux with stirring for 6 h yielded a compound which was identified by its lattice parameters as that one reported earlier (Starosta & Leciejewicz, 2010). After an addition of few drops of hydrazine, its warm aqueous solution was stirred for two h without heating. Left to crystallize at room temperature, single-crystal plates of the title compound were found after a couple of days. They were washed with cold ethanol and dried in air.

### Refinement

Coordinated water, hydrazine hydrogen atoms and crystal water molecule were located in a difference map and refined isotropically, while H atoms attached to pyridazine-ring C atoms were located at calculated positions and treated as riding on the parent atoms with C—H=0.93 Å and *U*_iso_(H)=1.2*U*_eq_(C).

### Crystal data

**Table:**

(N_2_H_5_)[Li_3_(C_6_H_2_N_2_O_4_)_2_(H_2_O)_2_]·H_2_O	*Z* = 2
*M**_r_* = 440.12	*F*(000) = 452
Triclinic, *P*1	*D*_x_ = 1.615 Mg m^−^^3^
Hall symbol: -P 1	Mo *K*α radiation, λ = 0.71073 Å
*a* = 5.215 (1) Å	Cell parameters from 25 reflections
*b* = 7.3356 (15) Å	θ = 6–15°
*c* = 24.001 (5) Å	µ = 0.14 mm^−^^1^
α = 97.62 (3)°	*T* = 293 K
β = 90.62 (3)°	Plate, colourless
γ = 95.77 (3)°	0.40 × 0.14 × 0.06 mm
*V* = 905.2 (3) Å^3^	

### Data collection

**Table:**

Kuma KM-4 four-cricle diffractometer	2660 reflections with *I* > 2σ(*I*)
Radiation source: fine-focus sealed tube	*R*_int_ = 0.051
Graphite monochromator	θ_max_ = 30.1°, θ_min_ = 1.7°
profile data from ω/2θ scans	*h* = −7→0
Absorption correction: analytical (*CrysAlis RED*; Oxford Diffraction, 2008)	*k* = −9→10
*T*_min_ = 0.982, *T*_max_ = 0.992	*l* = −31→33
5141 measured reflections	3 standard reflections every 200 reflections
4676 independent reflections	intensity decay: 1.4%

### Refinement

**Table:**

Refinement on *F*^2^	Primary atom site location: structure-invariant direct methods
Least-squares matrix: full	Secondary atom site location: difference Fourier map
*R*[*F*^2^ > 2σ(*F*^2^)] = 0.057	Hydrogen site location: inferred from neighbouring sites
*wR*(*F*^2^) = 0.243	H atoms treated by a mixture of independent and constrained refinement
*S* = 1.06	*w* = 1/[σ^2^(*F*_o_^2^) + (0.1704*P*)^2^ + 0.0007*P*] where *P* = (*F*_o_^2^ + 2*F*_c_^2^)/3
4676 reflections	(Δ/σ)_max_ < 0.001
329 parameters	Δρ_max_ = 0.56 e Å^−^^3^
10 restraints	Δρ_min_ = −0.48 e Å^−^^3^

### Special details

**Table:**

Geometry. All e.s.d.'s (except the e.s.d. in the dihedral angle between two l.s. planes) are estimated using the full covariance matrix. The cell e.s.d.'s are taken into account individually in the estimation of e.s.d.'s in distances, angles and torsion angles; correlations between e.s.d.'s in cell parameters are only used when they are defined by crystal symmetry. An approximate (isotropic) treatment of cell e.s.d.'s is used for estimating e.s.d.'s involving l.s. planes.
Refinement. Refinement of *F*^2^ against ALL reflections. The weighted *R*-factor *wR* and goodness of fit *S* are based on *F*^2^, conventional *R*-factors *R* are based on *F*, with *F* set to zero for negative *F*^2^. The threshold expression of *F*^2^ > σ(*F*^2^) is used only for calculating *R*-factors(gt) *etc*. and is not relevant to the choice of reflections for refinement. *R*-factors based on *F*^2^ are statistically about twice as large as those based on *F*, and *R*- factors based on ALL data will be even larger.

### Fractional atomic coordinates and isotropic or equivalent isotropic displacement parameters (Å^2^)

**Table:**

	*x*	*y*	*z*	*U*_iso_*/*U*_eq_	
O21	0.5659 (4)	0.6958 (3)	−0.10200 (9)	0.0309 (5)	
O12	0.1688 (4)	0.6942 (3)	0.56969 (10)	0.0294 (5)	
N21	0.7101 (4)	0.8483 (3)	0.00022 (10)	0.0205 (5)	
O11	0.5736 (4)	0.7887 (3)	0.60076 (9)	0.0306 (5)	
N11	0.7993 (5)	0.8737 (3)	0.44798 (10)	0.0227 (5)	
O23	0.6818 (5)	0.9204 (3)	0.19576 (9)	0.0301 (5)	
O22	0.1624 (4)	0.6266 (3)	−0.07499 (10)	0.0307 (5)	
N12	0.7115 (5)	0.8489 (3)	0.49847 (10)	0.0219 (5)	
O24	0.9860 (5)	1.0790 (3)	0.15156 (9)	0.0335 (5)	
C23	0.6641 (5)	0.8841 (4)	0.09640 (12)	0.0215 (6)	
C17	0.4028 (5)	0.7428 (4)	0.56305 (12)	0.0218 (5)	
N22	0.7999 (5)	0.9209 (3)	0.05164 (10)	0.0225 (5)	
C28	0.7876 (6)	0.9699 (4)	0.15267 (13)	0.0255 (6)	
C18	0.7881 (6)	0.8250 (4)	0.34710 (12)	0.0258 (6)	
O13	0.9742 (5)	0.9479 (3)	0.34981 (10)	0.0368 (6)	
C16	0.6660 (6)	0.7916 (4)	0.40213 (12)	0.0219 (6)	
C27	0.3945 (5)	0.6834 (4)	−0.06642 (12)	0.0225 (6)	
C14	0.3368 (6)	0.6633 (4)	0.45630 (12)	0.0244 (6)	
H14	0.1787	0.5962	0.4605	0.029*	
C24	0.4272 (6)	0.7763 (4)	0.09217 (13)	0.0261 (6)	
H24	0.3370	0.7524	0.1241	0.031*	
C26	0.4807 (5)	0.7464 (4)	−0.00552 (12)	0.0199 (5)	
C25	0.3319 (5)	0.7071 (4)	0.03952 (13)	0.0259 (6)	
H25	0.1732	0.6362	0.0342	0.031*	
C13	0.4859 (5)	0.7499 (4)	0.50274 (12)	0.0198 (5)	
C15	0.4332 (6)	0.6814 (4)	0.40447 (13)	0.0275 (6)	
H15	0.3468	0.6225	0.3719	0.033*	
Li1	1.0824 (10)	1.0999 (8)	0.4223 (2)	0.0296 (11)	
Li2	0.9101 (9)	0.8297 (8)	−0.0796 (2)	0.0283 (11)	
Li3	0.7103 (10)	0.6895 (7)	0.2248 (2)	0.0278 (11)	
N1	0.4289 (6)	0.1897 (5)	0.27436 (13)	0.0376 (7)	
N2	0.1607 (6)	0.1293 (5)	0.26487 (13)	0.0404 (7)	
O1	0.4489 (5)	0.4788 (4)	0.20777 (11)	0.0344 (6)	
H11	0.449 (10)	0.404 (7)	0.230 (2)	0.052*	
H12	0.449 (9)	0.426 (7)	0.170 (2)	0.052*	
O3	0.9495 (6)	0.4090 (4)	0.32720 (12)	0.0462 (7)	
H31	0.912 (10)	0.399 (8)	0.3605 (11)	0.069*	
H32	0.852 (9)	0.474 (7)	0.3144 (19)	0.069*	
O2	0.9871 (5)	0.5947 (4)	0.18013 (13)	0.0465 (7)	
O14	0.6997 (5)	0.7239 (4)	0.30427 (10)	0.0383 (6)	
H22	0.977 (14)	0.524 (8)	0.1501 (17)	0.10 (2)*	
H21	1.112 (9)	0.559 (8)	0.195 (3)	0.08 (2)*	
H1	0.451 (11)	0.188 (7)	0.3107 (9)	0.064 (16)*	
H4	0.138 (13)	0.029 (6)	0.283 (2)	0.081 (18)*	
H2	0.497 (9)	0.104 (5)	0.2505 (18)	0.058 (14)*	
H5	0.066 (12)	0.221 (7)	0.280 (3)	0.11 (3)*	
H3	0.124 (12)	0.102 (8)	0.2283 (10)	0.082 (19)*	

### Atomic displacement parameters (Å^2^)

**Table:**

	*U*^11^	*U*^22^	*U*^33^	*U*^12^	*U*^13^	*U*^23^
O21	0.0201 (10)	0.0448 (13)	0.0251 (11)	−0.0021 (9)	0.0015 (8)	−0.0012 (9)
O12	0.0152 (10)	0.0384 (12)	0.0354 (12)	0.0010 (8)	0.0057 (8)	0.0091 (9)
N21	0.0150 (11)	0.0246 (11)	0.0215 (12)	0.0002 (8)	0.0003 (8)	0.0029 (9)
O11	0.0194 (10)	0.0481 (13)	0.0235 (11)	−0.0024 (9)	0.0014 (8)	0.0065 (9)
N11	0.0177 (11)	0.0273 (12)	0.0236 (12)	−0.0010 (9)	0.0000 (9)	0.0071 (9)
O23	0.0348 (12)	0.0328 (11)	0.0240 (11)	0.0046 (9)	0.0060 (9)	0.0071 (8)
O22	0.0164 (10)	0.0389 (12)	0.0344 (12)	−0.0016 (8)	−0.0022 (8)	−0.0004 (9)
N12	0.0168 (11)	0.0272 (12)	0.0220 (12)	0.0019 (9)	0.0019 (9)	0.0039 (9)
O24	0.0317 (13)	0.0422 (13)	0.0237 (11)	−0.0107 (10)	−0.0029 (9)	0.0048 (9)
C23	0.0184 (13)	0.0228 (13)	0.0240 (13)	0.0029 (10)	0.0022 (10)	0.0046 (10)
C17	0.0192 (13)	0.0234 (13)	0.0236 (13)	0.0044 (10)	0.0031 (10)	0.0041 (10)
N22	0.0165 (11)	0.0266 (12)	0.0239 (12)	0.0008 (9)	0.0005 (9)	0.0026 (9)
C28	0.0259 (15)	0.0251 (13)	0.0262 (15)	0.0050 (11)	0.0014 (11)	0.0036 (11)
C18	0.0274 (15)	0.0281 (14)	0.0225 (14)	0.0027 (11)	0.0033 (11)	0.0061 (11)
O13	0.0379 (13)	0.0428 (13)	0.0266 (12)	−0.0125 (10)	0.0073 (10)	0.0053 (9)
C16	0.0213 (13)	0.0231 (13)	0.0209 (13)	0.0013 (10)	0.0003 (10)	0.0029 (10)
C27	0.0181 (13)	0.0237 (13)	0.0257 (14)	0.0037 (10)	−0.0019 (10)	0.0016 (10)
C14	0.0182 (13)	0.0282 (14)	0.0262 (14)	−0.0020 (10)	−0.0003 (10)	0.0047 (11)
C24	0.0195 (14)	0.0326 (15)	0.0269 (15)	−0.0001 (11)	0.0056 (11)	0.0083 (11)
C26	0.0130 (12)	0.0209 (12)	0.0262 (14)	0.0049 (9)	0.0010 (10)	0.0030 (10)
C25	0.0148 (13)	0.0294 (14)	0.0329 (16)	−0.0023 (10)	0.0021 (11)	0.0060 (12)
C13	0.0146 (12)	0.0234 (12)	0.0226 (13)	0.0047 (9)	0.0030 (9)	0.0054 (9)
C15	0.0225 (14)	0.0310 (15)	0.0263 (15)	−0.0040 (11)	−0.0045 (11)	0.0000 (11)
Li1	0.017 (2)	0.037 (3)	0.034 (3)	−0.002 (2)	0.000 (2)	0.004 (2)
Li2	0.017 (2)	0.038 (3)	0.029 (3)	0.001 (2)	0.0007 (19)	0.002 (2)
Li3	0.030 (3)	0.034 (3)	0.018 (2)	−0.002 (2)	0.0002 (19)	0.0032 (19)
N1	0.0311 (15)	0.0461 (17)	0.0335 (16)	−0.0010 (12)	−0.0028 (12)	0.0021 (13)
N2	0.0354 (17)	0.054 (2)	0.0300 (16)	−0.0107 (14)	−0.0003 (12)	0.0093 (14)
O1	0.0334 (13)	0.0375 (13)	0.0298 (13)	−0.0042 (10)	0.0006 (10)	0.0012 (10)
O3	0.0443 (16)	0.0570 (17)	0.0426 (15)	0.0141 (13)	0.0074 (12)	0.0186 (13)
O2	0.0270 (14)	0.0605 (18)	0.0490 (17)	0.0069 (12)	0.0068 (12)	−0.0063 (14)
O14	0.0470 (15)	0.0435 (14)	0.0210 (11)	−0.0076 (11)	−0.0008 (10)	0.0006 (9)

### Geometric parameters (Å, º)

**Table:**

O21—C27	1.248 (4)	C24—H24	0.9300
O12—C17	1.254 (3)	C26—C25	1.382 (4)
O12—Li1^i^	2.088 (6)	C25—H25	0.9300
N21—N22	1.337 (3)	C15—H15	0.9300
N21—C26	1.341 (3)	Li1—N11	2.260 (6)
O11—C17	1.252 (4)	Li1—O13	1.982 (6)
O11—Li1^ii^	2.010 (6)	Li1—O11^ii^	2.010 (5)
N11—N12	1.329 (3)	Li1—O12^i^	2.088 (6)
N11—C16	1.334 (4)	Li1—N12^ii^	2.144 (6)
O23—C28	1.256 (4)	Li2—O21	1.990 (6)
O22—C27	1.246 (4)	Li2—N21	2.186 (6)
O22—Li2^iii^	2.097 (6)	Li2—O24^iv^	1.995 (6)
N12—C13	1.331 (4)	Li2—O22^v^	2.097 (6)
N12—Li1^ii^	2.144 (6)	Li2—N22^iv^	2.276 (6)
O24—C28	1.245 (4)	Li3—O14	1.893 (5)
O24—Li2^iv^	1.995 (6)	Li3—O2	1.938 (6)
C23—N22	1.335 (4)	Li3—O1	1.952 (6)
C23—C24	1.393 (4)	Li3—O23	1.934 (6)
C23—C28	1.520 (4)	N1—N2	1.430 (4)
C17—C13	1.521 (4)	N1—H1	0.88 (2)
N22—Li2^iv^	2.276 (6)	N1—H2	0.897 (19)
C18—O14	1.240 (4)	N2—H4	0.90 (2)
C18—O13	1.251 (4)	N2—H5	0.92 (2)
C18—C16	1.511 (4)	N2—H3	0.89 (2)
C16—C15	1.396 (4)	O1—H11	0.82 (5)
C27—C26	1.522 (4)	O1—H12	0.93 (5)
C14—C15	1.364 (4)	O3—H31	0.835 (19)
C14—C13	1.395 (4)	O3—H32	0.814 (19)
C14—H14	0.9300	O2—H22	0.83 (2)
C24—C25	1.367 (4)	O2—H21	0.82 (2)
			
C27—O21—Li2	120.1 (3)	N12—C13—C14	123.2 (2)
C17—O12—Li1^i^	117.5 (2)	N12—C13—C17	113.8 (2)
N22—N21—C26	119.4 (2)	C14—C13—C17	122.9 (2)
N22—N21—Li2	129.1 (2)	C14—C15—C16	117.6 (3)
C26—N21—Li2	110.7 (2)	C14—C15—H15	121.2
C17—O11—Li1^ii^	117.2 (2)	C16—C15—H15	121.2
N12—N11—C16	119.5 (2)	O13—Li1—O11^ii^	98.3 (3)
N12—N11—Li1	129.9 (2)	O13—Li1—O12^i^	103.8 (3)
C16—N11—Li1	108.1 (2)	O11^ii^—Li1—O12^i^	108.2 (3)
C28—O23—Li3	126.2 (3)	O13—Li1—N12^ii^	153.2 (3)
C27—O22—Li2^iii^	116.2 (2)	O11^ii^—Li1—N12^ii^	79.1 (2)
N11—N12—C13	119.7 (2)	O12^i^—Li1—N12^ii^	102.3 (2)
N11—N12—Li1^ii^	128.2 (2)	O13—Li1—N11	76.6 (2)
C13—N12—Li1^ii^	110.9 (2)	O11^ii^—Li1—N11	155.5 (3)
C28—O24—Li2^iv^	119.8 (2)	O12^i^—Li1—N11	96.3 (2)
N22—C23—C24	123.0 (3)	N12^ii^—Li1—N11	94.7 (2)
N22—C23—C28	114.7 (2)	O21—Li2—O24^iv^	100.7 (3)
C24—C23—C28	122.3 (2)	O21—Li2—O22^v^	106.4 (3)
O11—C17—O12	126.9 (3)	O24^iv^—Li2—O22^v^	101.2 (2)
O11—C17—C13	116.8 (2)	O21—Li2—N21	77.79 (19)
O12—C17—C13	116.3 (3)	O24^iv^—Li2—N21	153.1 (3)
C23—N22—N21	119.3 (2)	O22^v^—Li2—N21	105.0 (3)
C23—N22—Li2^iv^	107.7 (2)	O21—Li2—N22^iv^	156.5 (3)
N21—N22—Li2^iv^	130.4 (2)	O24^iv^—Li2—N22^iv^	76.4 (2)
O24—C28—O23	126.5 (3)	O22^v^—Li2—N22^iv^	97.0 (2)
O24—C28—C23	117.1 (3)	N21—Li2—N22^iv^	94.3 (2)
O23—C28—C23	116.5 (3)	O14—Li3—O23	110.5 (3)
O14—C18—O13	126.7 (3)	O14—Li3—O2	126.1 (3)
O14—C18—C16	116.8 (3)	O23—Li3—O2	101.0 (3)
O13—C18—C16	116.4 (3)	O14—Li3—O1	99.9 (3)
C18—O13—Li1	120.8 (2)	O23—Li3—O1	121.7 (3)
N11—C16—C15	122.9 (3)	O2—Li3—O1	99.0 (3)
N11—C16—C18	114.9 (2)	N2—N1—H1	103 (4)
C15—C16—C18	122.2 (3)	N2—N1—H2	100 (3)
O22—C27—O21	127.7 (3)	H1—N1—H2	118 (5)
O22—C27—C26	116.6 (3)	N1—N2—H4	103 (4)
O21—C27—C26	115.8 (2)	N1—N2—H5	109 (5)
C15—C14—C13	117.0 (3)	H4—N2—H5	112 (6)
C15—C14—H14	121.5	N1—N2—H3	111 (4)
C13—C14—H14	121.5	H4—N2—H3	112 (6)
C25—C24—C23	117.7 (3)	H5—N2—H3	109 (6)
C25—C24—H24	121.2	Li3—O1—H11	114 (4)
C23—C24—H24	121.2	Li3—O1—H12	113 (3)
N21—C26—C25	123.3 (3)	H11—O1—H12	113 (5)
N21—C26—C27	113.8 (2)	H31—O3—H32	109 (3)
C25—C26—C27	122.9 (2)	Li3—O2—H22	129 (5)
C24—C25—C26	117.4 (3)	Li3—O2—H21	121 (5)
C24—C25—H25	121.3	H22—O2—H21	100 (6)
C26—C25—H25	121.3	C18—O14—Li3	143.4 (3)

Symmetry codes: (i) −*x*+1, −*y*+2, −*z*+1; (ii) −*x*+2, −*y*+2, −*z*+1; (iii) *x*−1, *y*, *z*; (iv) −*x*+2, −*y*+2, −*z*; (v) *x*+1, *y*, *z*.

### Hydrogen-bond geometry (Å, º)

**Table:**

*D*—H···*A*	*D*—H	H···*A*	*D*···*A*	*D*—H···*A*
N2—H3···O24^vi^	0.89 (2)	1.95 (2)	2.824 (4)	168 (6)
N2—H5···O3^iii^	0.92 (2)	1.82 (3)	2.709 (5)	162 (7)
N1—H2···O23^vii^	0.90 (2)	2.07 (2)	2.965 (4)	175 (5)
N2—H4···O13^vi^	0.90 (2)	1.96 (5)	2.716 (4)	140 (6)
N1—H1···O11^viii^	0.88 (2)	2.12 (2)	2.981 (4)	167 (5)
O2—H21···O1^v^	0.82 (2)	1.94 (3)	2.737 (4)	163 (7)
O2—H22···O22^ix^	0.83 (2)	2.07 (3)	2.868 (4)	162 (7)
O3—H31···O12^viii^	0.84 (2)	1.93 (2)	2.741 (4)	164 (5)
O3—H32···O14	0.81 (2)	2.11 (3)	2.875 (4)	156 (5)
O1—H12···O21^ix^	0.93 (5)	1.76 (5)	2.684 (3)	174 (4)
O1—H11···N1	0.82 (5)	2.00 (5)	2.814 (4)	171 (5)

Symmetry codes: (iii) *x*−1, *y*, *z*; (v) *x*+1, *y*, *z*; (vi) *x*−1, *y*−1, *z*; (vii) *x*, *y*−1, *z*; (viii) −*x*+1, −*y*+1, −*z*+1; (ix) −*x*+1, −*y*+1, −*z*.
